# Association of NF-κB and AP-1 with MMP-9 Overexpression in 2-Chloroethanol Exposed Rat Astrocytes

**DOI:** 10.3390/cells7080096

**Published:** 2018-08-07

**Authors:** Tong Wang, Xiaoxia Jin, Yingjun Liao, Qi Sun, Chaohong Luo, Gaoyang Wang, Fenghong Zhao, Yaping Jin

**Affiliations:** 1 Department of Occupational and Environmental Health, School of Public Health, China Medical University, No. 77 Puhe Road, Shenyang North New Area, Shenyang 110122, Liaoning, China; wang1234.sm@163.com (T.W.); xxjincmu@163.com (X.J.); sunqi@cmu.edu.cn (Q.S.); 13940522351@163.com (C.L.); gywang@cmu.edu.cn (G.W.); fhzhao@cmu.edu.cn (F.Z.); 2 Department of Physiology, China Medical University, Shenyang 110122, Liaoning, China; yjliao@cmu.edu.cn

**Keywords:** 1,2-Dichloroethane poisoning, 2-Chloroethanol, matrix metalloproteinases-9, p38 MAPK signal pathway, nuclear factor-κB, activator protein-1

## Abstract

Subacute poisoning of 1,2-dichloroethane (1,2-DCE) has become a serious occupational problem in China, and brain edema is its main pathological consequence, but little is known about the underlying mechanisms. As the metabolite of 1,2-DCE, 2-chloroethanol (2-CE) is more reactive, and might play an important role in the toxic effects of 1,2-DCE. In our previous studies, we found that matrix metalloproteinases-9 (MMP-9) expression was enhanced in mouse brains upon treatment with 1,2-DCE, and in rat astrocytes exposed to 2-CE. In the present study, we analyzed the association of nuclear factor kappa B (NF-κB) and activator protein-1 (AP-1) with MMP-9 overexpression in astrocytes treated with 2-CE. MMP-9, p65, c-Jun, and c-Fos were significantly upregulated by 2-CE treatment, which also enhanced phosphorylation of c-Jun, c-Fos and inhibitor of κBα (IκBα), and nuclear translocation of p65. Furthermore, inhibition of IκBα phosphorylation and AP-1 activity with the specific inhibitors could attenuate MMP-9 overexpression in the cells. On the other hand, inhibition of p38 mitogen-activated protein kinase (p38 MAPK) signaling pathway suppressed the activation of both NF-κB and AP-1 in 2-CE-treated astrocytes. In conclusion, MMP-9 overexpression induced by 2-CE in astrocytes could be mediated at least in part through the p38 signaling pathway via activation of both NF-κB and AP-1. This study might provide novel clues for clarifying the mechanisms underlying 1,2-DCE associated cerebral edema.

## 1. Introduction

The synthetic halohydrocarbon 1,2-dichloroethane (1,2-DCE) is mainly used as the monomer in the manufacture of polyvinyl chloride, and as an industrial solvent and glue thinner. It presents a significant occupational hazard to factory workers as exposure to high concentrations of 1,2-DCE vapors is lethal. In the last thirty years, a number of cases of subacute poisoning of 1,2-DCE have been reported in China [1]. Brain edema is the main pathological consequence of 1,2-DCE poisoning [2,3], but little is known regarding the underlying mechanisms. 

Previous studies have shown that 2-chloroethanol (2-CE), a metabolite of 1,2-DCE generated in vivo via microsomal CYP2E1, is more reactive than its parent molecule, and therefore might play an important role in the toxic effects of 1,2-DCE [4,5,6]. Our studies showed that matrix metalloproteinases-9 (MMP-9) was transcriptionally upregulated in 2-CE-treated astrocytes in vitro [7], as well as in the early phase of brain edema induced by subacute 1,2-DCE poisoning in mice [8]. MMP-9 is a zinc-dependent endopeptidase that degrades components of the extracellular matrix (ECM) in both physiological and pathological processes [9]. Although MMP-9 is normally present at low levels in astrocytes, it can be dramatically enhanced by ischemic and hemorrhagic stroke, and is involved in subsequent brain injury and vasogenic brain edema [10,11]. Therefore, it is reasonable to assume that overexpression of MMP-9 plays an important mechanistic role in cerebral edema induced by 1,2-DCE. Accordingly, it is necessary to elucidate the pathways regulating MMP-9 overexpression in 2-CE-treated astrocytes. In our previous studies, the mitogen-activated protein kinase (MAPK) signaling pathways, including the extracellular signal-regulated kinase 1/2 (ERK 1/2), c-Jun amino terminal kinase (JNK), and p38 MAPK pathways, were shown to be involved in the upregulation of MMP-2 and MMP-9 in 2-CE-treated astrocytes [7,12]. In this study, we further explored the involvement of transcription factors in MMP-9 overexpression in 2-CE-treated astrocytes. 

Many reports have in fact indicated important roles of the transcription factors in regulating MMP-9 expression. The promoter region of the MMP-9 gene harbors binding sites for both nuclear factor-κB (NF-κB) and activator protein-1 (AP-1), and activation of both transcription factors is essential for MMP-9 expression [10,13,14,15,16]. However, whether they are also activated in 2-CE-treated astrocytes and drive MMP-9 overexpression in these cells is poorly understood. On the other hand, it is well-known that p38 MAPK plays an important role in the inflammation response, the main mechanism underlying brain edema. Therefore, to further investigate the regulatory pathways mediating MMP-9 overexpression induced by 2-CE in rat astrocytes, we treated the cells with specific inhibitors of NF-κB, AP-1 and p38 before 2-CE exposure. Our study might provide novel clues for clarifying the mechanisms underlying 1,2-DCE associated cerebral edema.

## 2. Materials and Methods

### 2.1. Reagents

The 2-Chloroethanol was purchased from Sinopharm Chemical Reagent Co., Ltd. (Ningbo, China). Reagents for the primary culture of astrocytes were purchased from Biological Industries (Beit-Haemek, Israel). The quantitative real-time (RT)-PCR assay kit was purchased from Takara, Japan. The enhanced chemiluminescence (ECL) plus kit, bicinchoninic acid (BCA) protein assay kit and NE-PER™ nuclear and cytoplasmic extraction reagents were obtained from Thermo Fisher Scientific (Waltham, MA, USA). SB202190, pyrrolidine dithiocarbamate (PDTC), and SR11302 were purchased from Selleck (Houston, TX, USA) and APExBIO (Houston, TX, USA). Primary antibodies against MMP-9, p65, IκBα, c-Jun, c-Fos, p-c-Jun, p-c-Fos, p-IκBα, and LaminB were products of Abcam (Cambridge, UK) and Cell Signaling Technology (Beverly, MA, USA). Antibodies against glial fibrillary acid protein (GFAP), glyceraldehyde 3-phosphate dehydrogenase (GAPDH), and β-actin were obtained from Millipore (Billerica, MA, USA), Proteintech (Wuhan, China), and ABclonal (Wuhan, China), respectively. The secondary antibodies conjugated with Alexa Fluor 488 or tetramethylrhodamine (TRITC), RIPA Lysis Buffer, and DAPI were obtained from Beyotime Biotechnology (Shanghai, China). 

### 2.2. Astrocyte Enrichment and Culture

All experiments were approved by the Animal Care and Use Committee at China Medical University, which complies with the recommendations of the Chinese National Guidelines for the Protection of Laboratory Animals. The project identification code was IACUC: NO.16101. The cerebral cortexes of neonatal rats were isolated and enzymatically digested [7,12]. The dissociated brain cells were then collected and plated in a culture dish pre-coated with poly-L-lysine. The cells were cultured in a humidified incubator at 37 °C under 5% CO_2_ until they formed a confluent layer. The oligodendrocytes and microglia were removed by shaking the culture dishes at 250 rpm for 15 h on a horizontal shaker. The remaining cells were resuspended and re-plated for further enrichment of the astrocytes, which were identified by positive immunostaining for the glial fibrillary acid protein (GFAP). The cultures with more than 95% astrocytes were used for subsequent experiments. 

### 2.3. In Vitro Treatments 

The 1 M stock solution of 2-CE was prepared with redistilled water, and then diluted to the final concentrations by Dulbecco’s Modified Eagle Medium (DMEM) containing 5% fetal bovine serum (FBS) before use. The primary astrocytes were treated with 30 mM 2-CE for 1, 2, 4, 8, 12, 24, and 48 h, and with 0, 7.5, 15, and 30 mM 2-CE for 12 h to determine the effect of different 2-CE concentrations and treatment durations on MMP-9 expression. To test the effects of NF-κB, AP-1, and p38 MAPK inhibition, astrocytes were pretreated with pyrrolidine dithiocarbamate (PDTC; 10, 25 µM for 2 h), SR11302 (5, 10 μM for 1 h), and SB202190 (1, 10, 30 μM for 1 h), respectively, before a 12 h treatment with 30 mM 2-CE. In addition, untreated controls, inhibitor controls, and solvent controls were also included. The different inhibitors were dissolved in water or dimethyl sulfoxide (DMSO) for preparing the stock solution, and then diluted by the culture media to the final dose. Solvent control cells were treated with 0.1% DMSO, and inhibitor controls with only PDTC (25 μM), SR11302 (10 μM), or SB202190 (30 μM). 

### 2.4. Immunofluorescence 

The cells were fixed and permeabilized as described [7], and then blocked with goat serum at room temperature. Thereafter, the cells were incubated overnight with rabbit anti-rat p65 and mouse anti-rat GFAP antibodies at 4 °C, followed by a 30 min incubation with goat anti-rabbit-Alexa Fluor 488 and goat anti-mouse TRITC secondary antibodies at 37 °C. The stained cells were observed under a fluorescence microscope (Olympus IX71, Tokyo, Japan), and imaged using a digital camera system (Olympus SC35, Tokyo, Japan). Negative controls lacking primary antibodies were also included.

### 2.5. Western Blotting

Total proteins were extracted with RIPA buffer, and the nuclear and cytoplasmic protein fractions were extracted with the NE-PER™ nuclear and cytoplasmic extraction reagents, respectively. Protein concentrations of the samples were determined by BCA protein assay kit. Equal amounts of sample proteins were separated by SDS-PAGE, and then transferred to a polyvinylidene difluoride (PVDF) membrane (Millipore, Burlington, MA, USA). The blots were incubated overnight with primary antibodies against MMP-9, c-Jun, c-Fos, IκBα, p65, p-c-Jun, p-c-Fos, p-IκBα, and GAPDH (internal control) at 4 °C. On the following day, the membranes were incubated with the secondary antibodies at room temperature for 1 h. The bands were imaged using Azure c300 Chemiluminescent Western Blot Imaging System (Azure Biosystems, Dublin, CA, USA) with the ECL Western blot chemiluminescent detection reagents. The intensity of the bands were analyzed semi-quantitatively via densitometry using the Gel-Pro analyzer v4.0 software (Meyer Instruments, Houston, TX, USA), and the relative protein expression levels were normalized to GAPDH.

### 2.6. Quantitative Real-Time (RT)-PCR

Total RNA was extracted using Trizol Reagent (Takara, Japan) and cDNA was synthesized by reverse transcription using PrimeScript RT reagent kit (Takara, Japan). MMP-9, p65, IκBα, c-Jun, c-Fos, and GAPDH (constitutive gene) fragments were then amplified using the specific primer pairs detailed in Table 1. The reaction was carried out for 40 cycles at 95 °C for 5 s and 60 °C for 34 s on a QuantStudio 6 Flex real-time PCR System (Life Technologies, Carlsbad, CA, USA) using the SYBR Premix Ex Taq II (Takara, Japan). The relative mRNA levels were analyzed using the comparative Ct method and expressed as 2^−ΔΔCt^ formula. GAPDH mRNA was used as an internal control for normalization of gene expression.

### 2.7. ELISA

Culture supernatants were centrifuged at 10,000 rpm for 5 min at 4 °C. Secreted MMP-9 levels in the supernatants were measured using the rat total MMP-9 ELISA Kit (Wuhan Boster Biological Technology Co., Ltd., Wuhan, China) according to the manufacturer’s instructions.

### 2.8. Statistical Analysis

The results are represented as the mean ± standard deviation (SD) of at least four independent experiments. Statistical analyses were carried out using SPSS for Windows, version 20.0 (SPSS, Armonk, NY, USA). Data were evaluated by one-way analysis of variance (ANOVA) followed by the Student–Newman–Keuls (SNK) test. A *p*-value of less than 0.05 was considered significant.

## 3. Results

### 3.1. Upregulated MMP-9 Expression in 2-CE Treated Rat Astrocytes along with the Exposure Duration and Concentrations

Although our previous study indicated that MMP-9 expression could be augmented in astrocytes upon 2-CE treatment, the transient expression profile of MMP-9 was unclear. Therefore, in this study, we tracked MMP-9 expression in the cells treated with 30 mM 2-CE for 1 h to 48 h.

**Figure 1:**
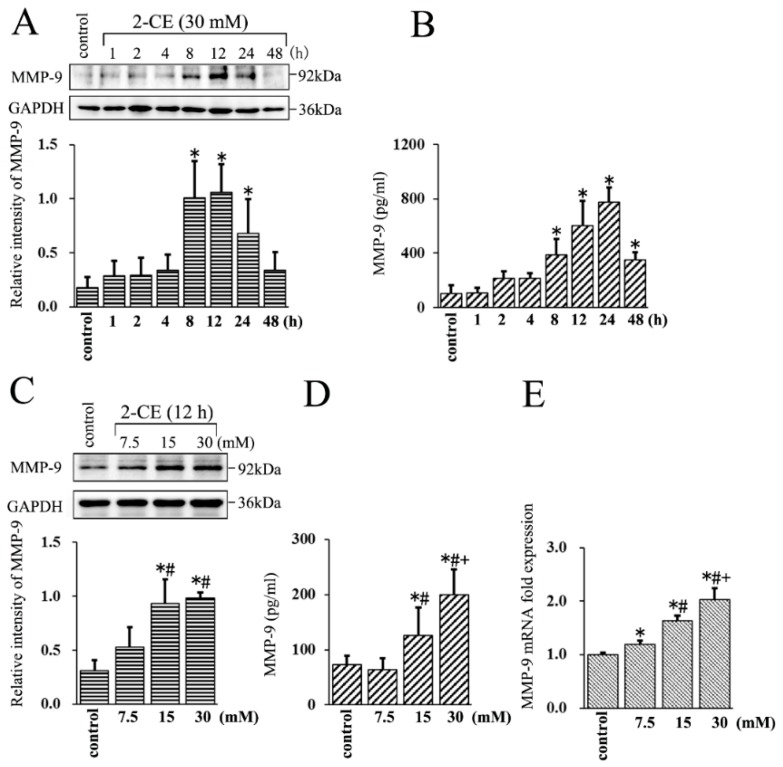
Effects of 2-chloroethanol (2-CE) on matrix metalloproteinases-9 (MMP-9) expression in astrocytes. (**A**,**B**) Astrocytes were treated with 30 mM 2-CE for the indicated times; (**C**–**E**) Astrocytes were treated with 0, 7.5, 15, and 30 mM 2-CE for 12 h. (**A**,**C**) MMP-9 protein levels in astrocytes measured by Western blotting; (**B**,**D**) MMP-9 protein levels in the culture media measured by ELISA; (**E**) MMP-9 mRNA levels in astrocytes analyzed by real-time (RT)-PCR. (*n* = 4; mean ± SD; * *p* < 0.05 vs. control group, ^#^ *p* < 0.05 vs. 7.5 mM of 2-CE, ^+^ *p* < 0.05 vs. 15 mM of 2-CE; one-way analysis of variance (ANOVA) followed by Student–Newman–Keuls (SNK) tests.).

The protein levels of MMP-9 increased in the astrocytes within 8 h of 2-CE exposure, and remained significantly above the control levels at 24 h (Figure 1A). Moreover, those in the culture media also increased significantly by 8 h, peaked at 24 h, and remained significantly above the control levels even after 48 h (Figure 1B). Therefore, in subsequent in vitro experiments, the cells were exposed to 2-CE for 12 h. In addition, the MMP-9 protein levels in both cells and culture media were significantly higher in the 15 and 30 mM groups compared with the control group or the 7.5 mM group, and were significantly higher in the culture media of the 30 mM group compared with the 15 mM group (Figure 1C,D). The MMP-9 mRNA levels also increased significantly upon 2-CE treatment in a dose dependent manner (Figure 1E).

### 3.2. Increased Expression and Nuclear Translocation of p65 in 2-CE Treated Rat Astrocytes along with the Exposure Duration and Concentrations

**Figure 2:**
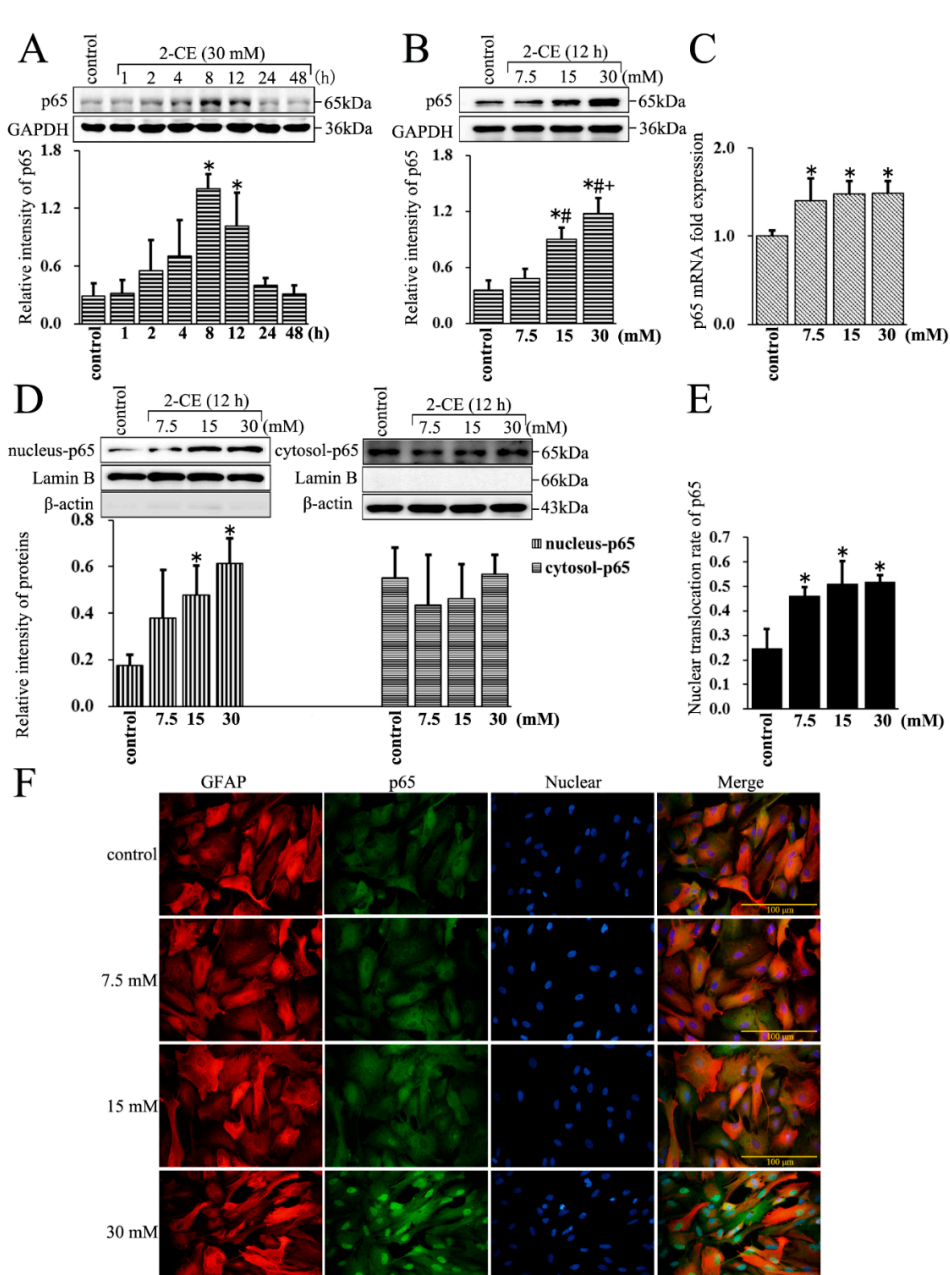
The nuclear translocation of p65 affected by 2-CE in astrocytes. (**A**) Astrocytes were treated with 30 mM of 2-CE for the indicated times, and (**B**–**F**) with 0, 7.5, 15, and 30 mM 2-CE for 12 h; (**A**,**B**) The levels of p65 in whole cell lysate measured by Western blotting; (**C**) The p65 mRNA levels analyzed by RT-PCR; (**D**) The cytosolic and nuclear p65 fractions measured by Western blotting, with β-actin and Lamin B as the respective internal standards; (**E**) The nuclear translocation rate of p65 was expressed as (relative intensity of p65 in nuclear fraction/[relative intensity of p65 in nuclear fraction + relative intensity of p65 in cytoplasmic fraction]); (**F**) Representative immunofluorescence images of p65 and glial fibrillary acid protein (GFAP) in primary culture astrocytes (400×). (*n* = 4; mean ± SD; * *p* < 0.05 vs. control group, ^#^ *p* < 0.05 vs. 7.5 mM of 2-CE, ^+^ *p* < 0.05 vs. 15 mM of 2-CE; one-way ANOVA followed by SNK tests.).

The protein levels of p65 increased in the whole cell lysate within 8 h of 2-CE exposure, and remained significantly above the control levels at 12 h (Figure 2A). Moreover, those were significantly higher in the 15 and 30 mM groups compared with the control group or 7.5 mM group, and were also significantly higher in the 30 mM group compared with the 15 mM group (Figure 2B). In addition, the p65 mRNA levels also increased with 2-CE treatment (Figure 2C). The nuclear p65 protein levels in the 15 and 30 mM 2-CE treated cells, and the nuclear translocation ratios of p65 in cells treated with every 2-CE dose, were significantly higher than in the untreated control cells (Figure 2D–F). However, 2-CE treatment did not have an obvious effect on cytosolic p65 protein levels.

### 3.3. Increased Expression and Phosphorylation of IκBα in 2-CE Treated Rat Astrocytes along with the Exposure Concentrations

**Figure 3:**
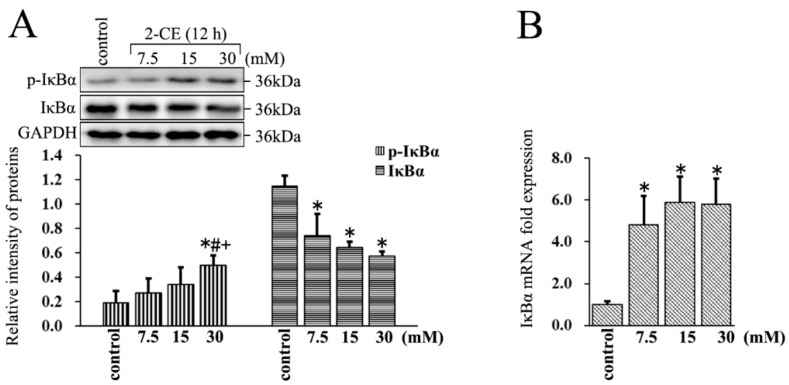
The phosphorylation of IκBα affected by 2-CE in astrocytes. (**A**,**B**) Astrocytes were treated with 0, 7.5, 15, and 30 mM 2-CE for 12 h; (**A**) The levels of p-inhibitor of κBα (IκBα) and IκBα in astrocytes measured by Western blotting; (**B**) The IκBα mRNA levels analyzed by RT-PCR. (*n* = 4; mean ± SD; * *p* < 0.05 vs. control group, ^#^ *p* < 0.05 vs. 7.5 mM of 2-CE, ^+^ *p* < 0.05 vs. 15 mM of 2-CE; one-way ANOVA followed by SNK tests.).

The IκBα protein levels decreased significantly in 2-CE treated astrocytes, whereas their IκBα mRNA levels increased significantly. Furthermore, the level of p-IκBα in the 30 mM group was significantly elevated compared with the control group and other exposure groups (Figure 3A,B). This clearly indicated that 2-CE treatment could enhance phosphorylation and degradation of IκBα, and activation of NF-κB in the astrocytes. In addition, our results also demonstrated that the transcriptional activation of IκBα was induced in 2-CE-treated astrocytes, which was probably due to IκBα degradation and NF-κB activation.

### 3.4. Upregulated Expression and Phosphorylation of c-Jun and c-Fos in 2-CE Treated Rat Astrocytes along with the Exposure Duration and Concentrations

**Figure 4:**
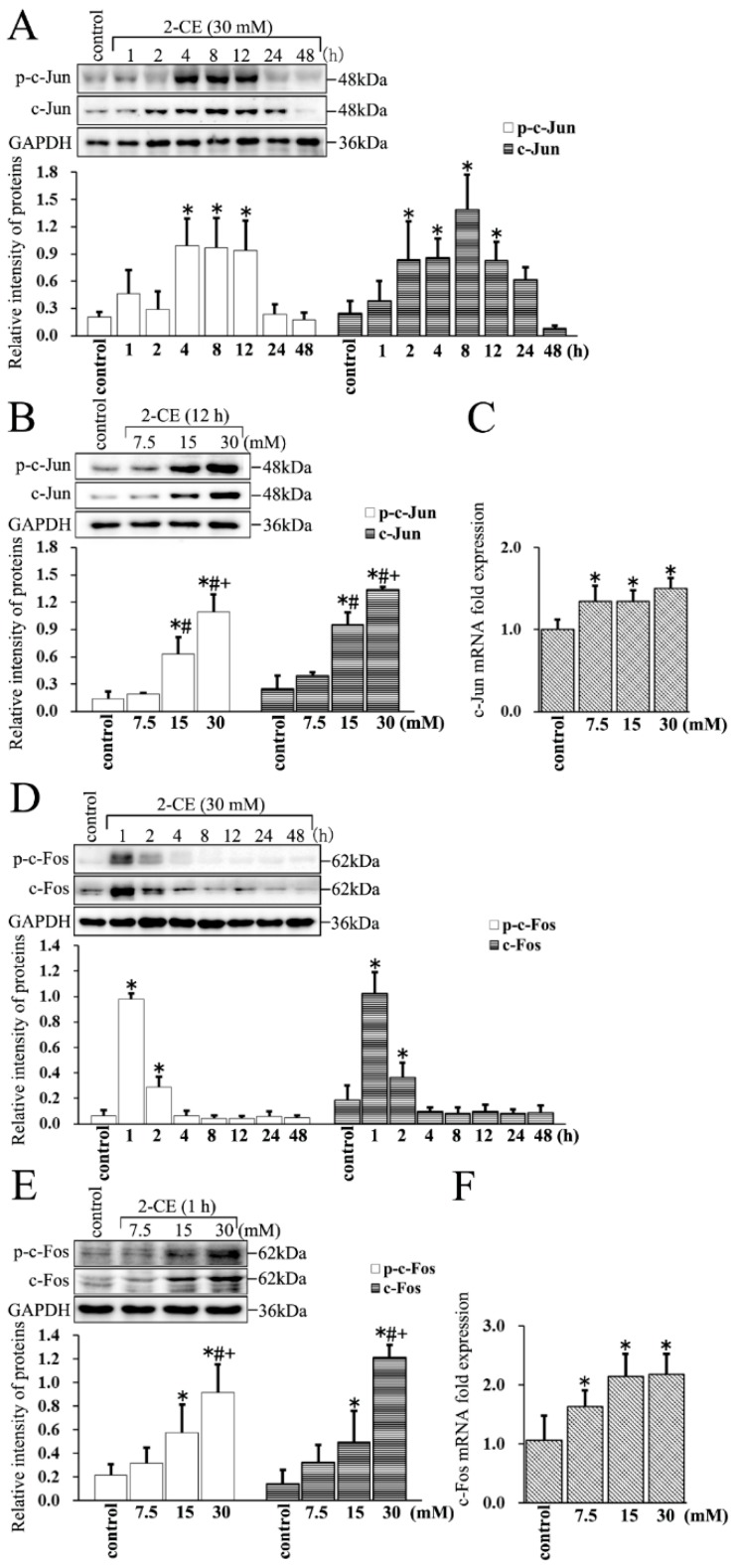
The expression and phosphorylation of c-Jun and c-Fos in astrocytes induced by 2-CE. (**A**,**D**) Astrocytes were treated with 30 mM 2-CE for the indicated times, and (**B**,**C**,**E**,**F**) with 0, 7.5, 15, and 30 mM 2-CE for 12 h; (**A**,**B**,**D**,**E**) Levels of p-c-Jun, p-c-Fos, c-Jun, and c-Fos in astrocytes measured by Western blotting; (**C**,**F**) The c-Jun and c-Fos mRNA levels analyzed by RT-PCR. (*n* = 4; mean ± SD; * *p* < 0.05 vs. control group, ^#^ *p* < 0.05 vs. 7.5 mM of 2-CE, ^+^ *p* < 0.05 vs. 15 mM of 2-CE; one-way ANOVA followed by SNK tests.).

The c-Jun protein levels increased significantly in 2-CE treated astrocytes as early as 2 h, peaked at 8 h, and remained significantly above the control levels at 12 h. The levels of p-c-Jun increased remarkably by 4 h, and remained significantly above the control levels at 12 h (Figure 4A). Furthermore, the levels of both c-Jun and p-c-Jun in 2-CE-treated cells increased significantly in a dose dependent manner (Figure 4B). In addition, the c-Jun mRNA levels in these cells also increased significantly (Figure 4C). Similarly, the levels of both c-Fos and p-c-Fos increased rapidly as early as 1 h, and remained significantly high at 2 h (Figure 4D). Therefore, to explore the changes in c-Fos expression due to different 2-CE concentrations, the astrocytes were treated with 2-CE for 1 h. Furthermore, the levels of c-Fos and p-c-Fos increased after treatment with 2-CE in a dose dependent manner (Figure 4E). Likewise, the mRNA levels of c-Fos in 2-CE-treated cells increased significantly (Figure 4F).

We therefore hypothesized that 2-CE induced overexpression and phosphorylation of c-Jun and c-Fos would activate AP-1 in the 2-CE-treated astrocytes. However, both c-Jun and c-Fos were overexpressed prior to their phosphorylation. In addition, as an immediate early gene, activation of c-Fos occurred earlier and was sustained for a very short time compared with c-Jun.

### 3.5. MMP-9 Overexpression Mediated by NF-κB and AP-1 in 2-CE-Treated Astrocytes

**Figure 5:**
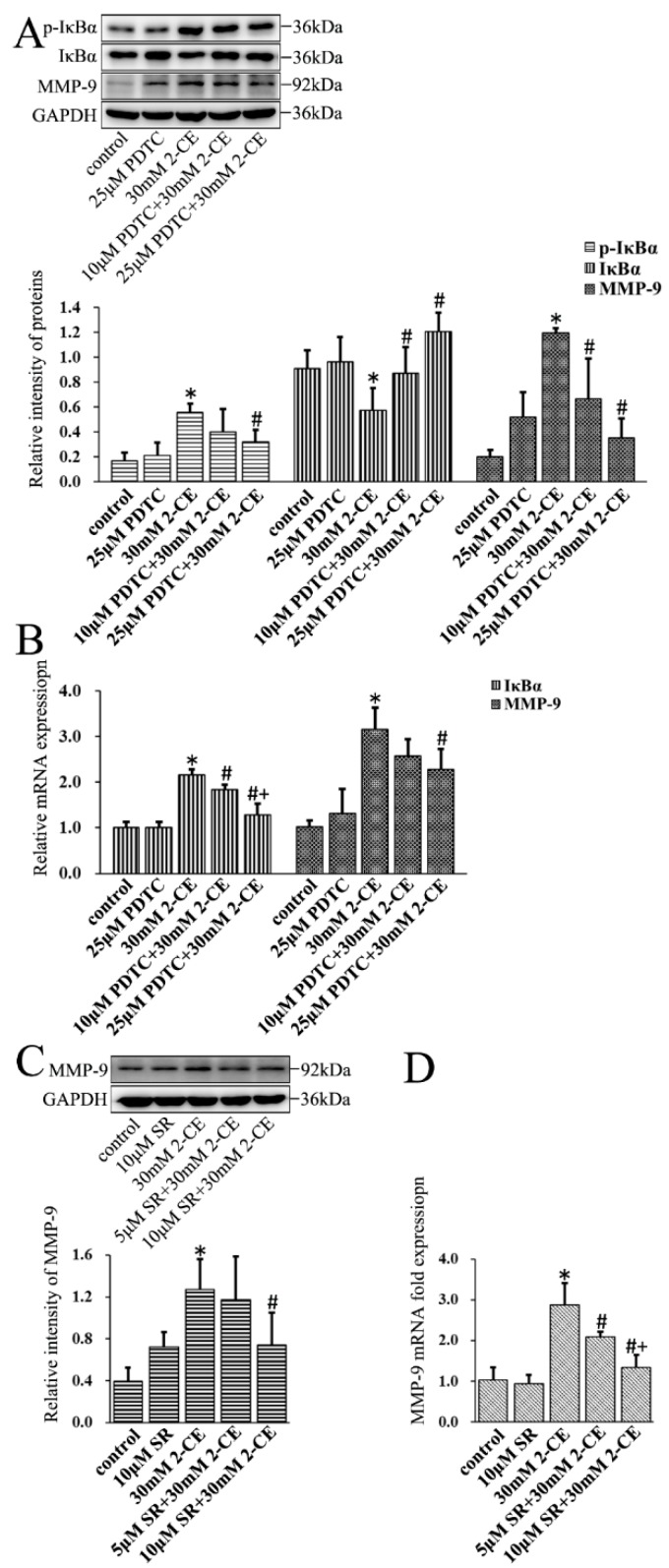
Role of nuclear factor kappa B (NF-κB) and activator protein-1 (AP-1) in 2-CE induced MMP-9 overexpression in astrocytes. (**A**,**B**) Astrocytes were pre-treated with 10 and 25 μM of pyrrolidine dithiocarbamate (PDTC) for 2 h and then treated with 30 mM 2-CE for 12 h; (**C**,**D**) Astrocytes were pre-treated with 5 and 10 μM SR for 1 h and then treated with 30 mM 2-CE for 12 h; (**A**,**C**) The levels of p-IκBα, IκBα, and MMP-9 in astrocytes measured by Western blotting; (**B**,**D**) The IκBα and MMP-9 mRNA levels analyzed by RT-PCR. (*n* = 4; mean ± SD; * *p* < 0.05 vs. control group, ^#^ *p* < 0.05 vs. 30 mM of 2-CE, ^+^ *p* < 0.05 vs. 10 μM of PDTC or 5 μM of SR; one-way ANOVA followed by SNK tests.).

To determine the role of NF-κB or AP-1 activation in MMP-9 overexpression mediated by 2-CE, the cells were pre-treated for 2 h with PDTC or 1 h with SR11302, specific inhibitors of IκBα phosphorylation and AP-1 activity, respectively, before a 12 h treatment with 30 mM 2-CE. Pre-treatment of astrocytes with 25 µM PDTC significantly lowered the levels of *p*-IκBα, while both 10 and 25 µM PDTC downregulated IκBα mRNA levels induced by 2-CE exposure. In contrast, both 10 and 25 µM PDTC restored the IκBα protein levels reduced by 2-CE treatment. As a result of the changes in p-IκBα and IκBα levels, MMP-9 expression was also significantly lower in cells pre-treated with PDTC compared with those treated only with 2-CE (Figure 5A,B).

Similarly, pre-treatment with SR11302 also downregulated MMP-9 expression in a dose dependent manner (Figure 5C,D).

### 3.6. Roles of p38 MAPK Signaling Pathway in NF-κB and AP-1 Activation in 2-CE Treated Astrocytes

**Figure 6:**
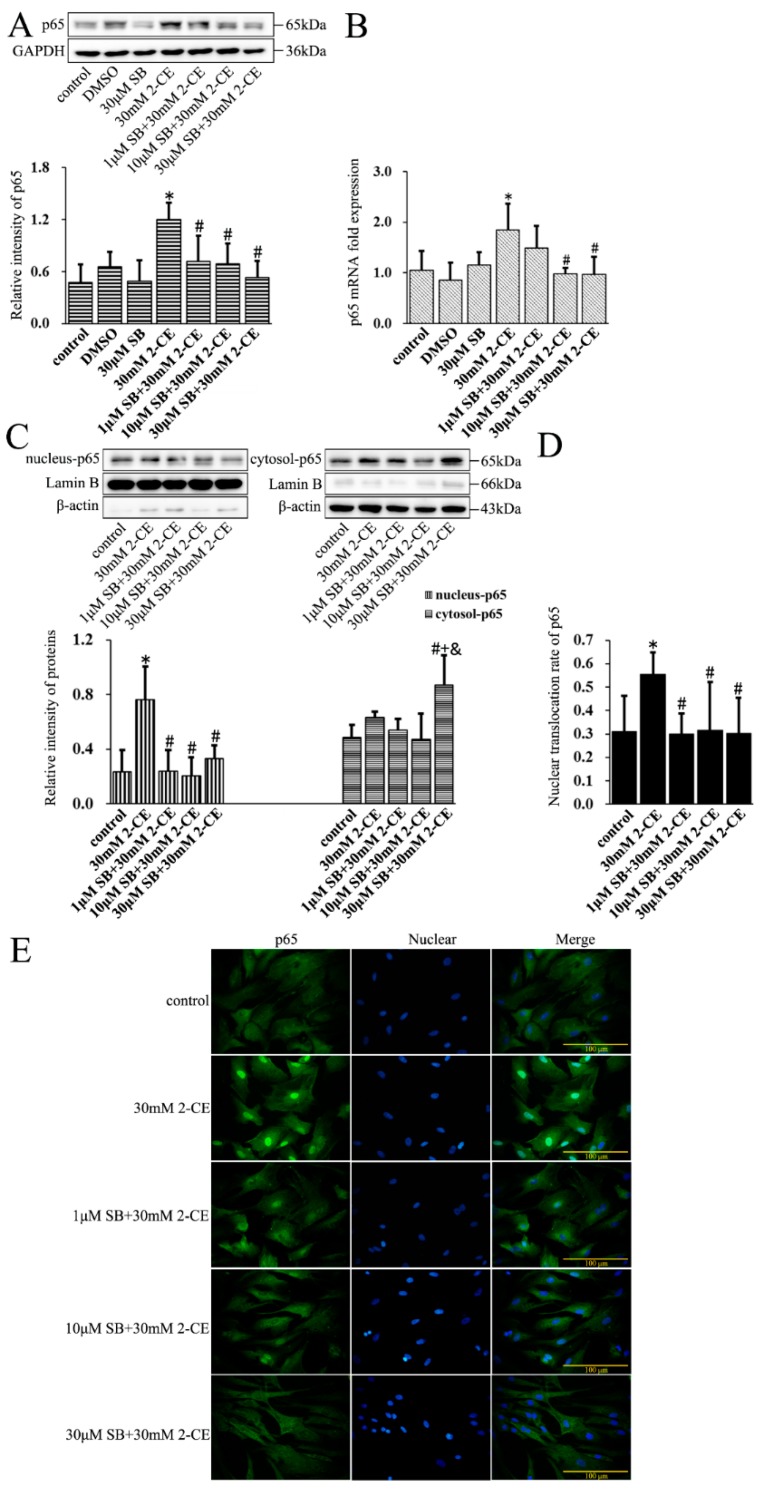
Role of p38 mitogen-activated protein kinase (p38 MAPK) signaling pathway in 2-CE induced nuclear translocation of p65 in astrocytes. Astrocytes were pre-treated with 1, 10, and 30 μM SB for 1 h and then treated with 30 mM of 2-CE for 12 h. (**A**) The levels of p65 in astrocytes measured by Western blotting; (**B**) The p65 mRNA levels analyzed by RT-PCR; (**C**) The cytosolic and nuclear fraction of p65 measured by Western blotting; (**D**) The nuclear translocation rate of p65; (**E**) Immunofluorescence staining for p65 (400×). (*n* = 4; mean ± SD; * *p* < 0.05 vs. control group, ^#^ *p* < 0.05 vs. 30 mM of 2-CE, ^+^ *p* < 0.05 vs. 1 μM of SB, ^&^ *p* < 0.05 vs. 10 μM of SB; one-way ANOVA followed by SNK tests.).

**Figure 7:**
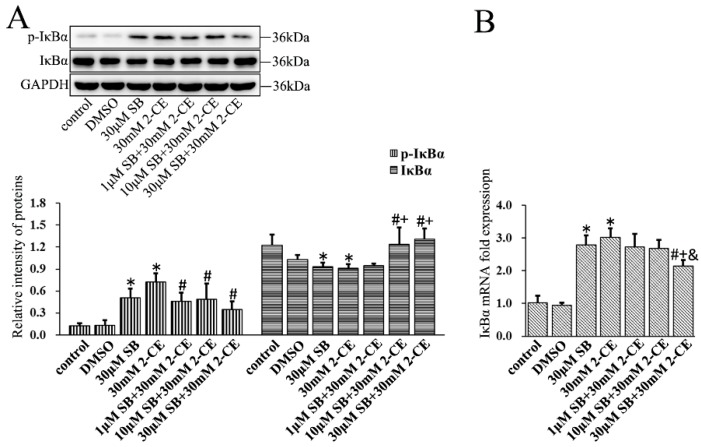
Role of p38 MAPK signaling pathway in 2-CE induced phosphorylation of IκBα in astrocytes. Astrocytes were pre-treated with 1, 10, and 30 μM SB for 1 h and then treated with 30 mM of 2-CE for 12 h. (**A**) The levels of *p*-IκBα and IκBα in astrocytes measured by Western blotting; (**B**) The IκBα mRNA levels were analyzed by RT-PCR. (*n* = 4; mean ± SD; * *p* < 0.05 vs. control group, ^#^ *p* < 0.05 vs. 30 mM of 2-CE, ^+^ *p* < 0.05 vs. 1 μM of SB, ^&^ *p* < 0.05 vs. 10 μM of SB; one-way ANOVA followed by SNK tests.).

To determine the association of the p38 MAPK signaling pathway with activating NF-κB and AP-1 in 2-CE treated astrocytes, cells were pre-treated for 1 h with SB202190, a specific inhibitor of p38 MAPK, before a 12 h treatment with 30 mM 2-CE. Inhibition of p38 MAPK significantly decreased the p65 protein and mRNA levels compared with the cells treated with 2-CE alone (Figure 6A,B). In addition, the level of nuclear p65 protein and its nuclear translocation ratio were decreased significantly upon pretreatment with 1, 10, and 30 μM SB202190, while the cytoplasmic p65 protein levels were significantly increased (Figure 6C–E). SB202190 also significantly decreased the levels of *p*-IκBα and IκBα mRNA, and increased IκBα protein levels. Surprisingly, treatment with SB202190 alone increased the phosphorylation and transcriptional expression of IκBα in astrocytes (Figure 7A,B).

**Figure 8:**
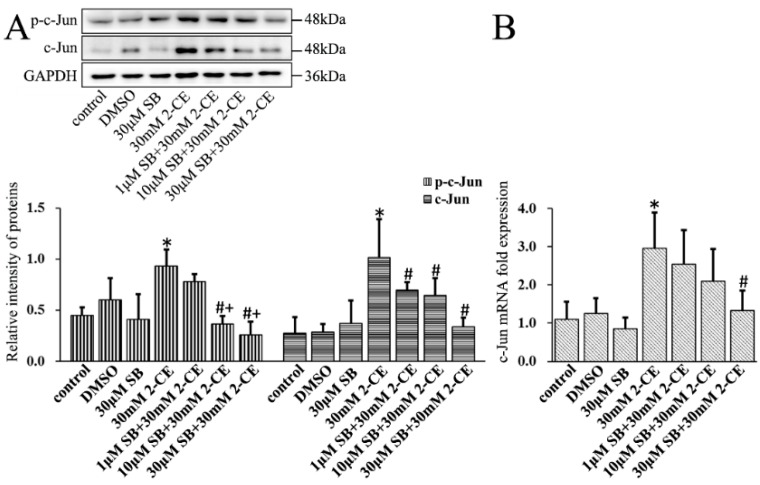
Role of p38 MAPK signal pathway in 2-CE induced activation of AP-1 in astrocytes. Astrocytes were pre-treated with 1, 10, and 30 μM SB for 1 h and then treated with 30 mM 2-CE for 12 h. (**A**) The levels of p-c-Jun and c-Jun in astrocytes measured by Western blotting; (**B**) The c-Jun mRNA levels analyzed by RT-PCR. (*n* = 4; mean ± SD; * *p* < 0.05 vs. control group, ^#^ *p* < 0.05 vs. 30 mM of 2-CE, ^+^ *p* < 0.05 vs. 1 μM of SB; one-way ANOVA followed by SNK tests.).

Finally, SB202190 mediated inhibition of p38 MAPK significantly decreased the levels of p-c-Jun and protein and mRNA levels of c-Jun in 2-CE-treated astrocytes (Figure 8). However, because c-Fos expression and phosphorylation occurred much earlier than the phosphorylation of p38 (data as shown in Figure S1), it is possible that activation of c-Fos might not be mediated by the p38 MAPK signaling pathway.

## 4. Discussion

Accumulated evidence has demonstrated that astrocytes are necessary for neuronal survival and function by maintenance of blood brain barrier (BBB) integrity and extracellular homeostasis. Astrocytes can be activated in pathophysiological conditions, and secrete a variety of proinflammatory cytokines, such as MMP-9, which then degrades the proteins in the tight junctions of BBB, thus leading to breakdown in BBB integrity and brain edema formation.

We previously investigated the association of MMP-9 overexpression with MAPK signaling pathways in 2-CE-treated rat astrocytes [7], but the transient expression of MMP-9 with the exposure duration was unclear. This point is significant because MMP-9 expression is inducible by this stimuli. To the best of our knowledge, this is the first report to show the transient expression profile of MMP-9 in either 2-CE-treated astrocytes or culture media. MMP-9 protein levels increased markedly in both astrocytes and their media within 8 h of 2-CE exposure, and remained high in the cells until 24 h, whereas high levels were detected in the culture media for 48 h, indicating that the overexpressed MMP-9 in astrocytes is simultaneously secreted. Furthermore, MMP-9 levels peaked at 12 h and 24 h in the astrocytes and culture media, respectively. As a result of a lack of MMP-9 specific proteases in the culture media, it could not be eliminated rapidly, and thus accumulated in the culture media. Therefore, MMP-9 was retained longer in the media, and peaked later than that in the astrocytes.

Recent studies have demonstrated that activation of MAPK signaling pathways can contribute to upregulation of MMP-9 expression in the astrocytes [17,18]. As overexpression of MMP-9 can result in breakdown of BBB in traumatic, hemorrhagic, and ischemic brain injury, studying the regulatory pathways is necessary for uncovering the mechanisms of 1,2-DCE induced brain edema. Although we also showed a positive association of MAPK signaling pathways with MMP-9 overexpression in 2-CE-treated astrocytes previously, we did not focus on the transcription factors involved. This is also the first report to show activation of NF-κB and AP-1 in 2-CE-treated astrocytes concomitant with MMP-9 overexpression. Both NF-κB and AP-1 are the key transcriptional regulators in pathways of inflammation, differentiation, proliferation, and apoptosis. Deregulation of NF-κB and AP-1 may result in the overexpression of downstream genes, including cytokines, chemokines, and effector proteins, and lead to cellular damage [19].

The NF-κB consists of five subunits in mammalian cells: c-Rel, RelA (p65), RelB, NF-κB1 (p50), and NF-κB2 (p52) [20]. The major form of NF-κB in cells is a heterodimer consisting of the DNA binding subunit p50 and the trans-activator p65 [21]. In quiescent cells, NF-κB is restricted to the cytoplasm by its binding with IκBα [22]. Upon stimulation by different upstream signals, IκBα is phosphorylated and degraded through an ubiquitin-dependent process, which releases NF-κB and allows its nuclear translocation to activate gene transcription [23,24]. Accordingly, as we found IκBα phosphorylation was enhanced in 2-CE-treated astrocytes, it could be speculated that decreased levels of IκBα protein and increased nuclear translocation of p65 might be the consequence of IκBα phosphorylation. In addition, p65 total protein and mRNA expression were also increased after 2-CE treatment. Furthermore, in response to suppressed IκBα phosphorylation, the overexpression of MMP-9 induced by 2-CE was ameliorated, indicating an involvement of NF-κB activation in MMP-9 overexpression mediated by 2-CE in astrocytes.

AP-1 consists of a set of structurally and functionally related members of the Jun protein family (c-Jun, JunB, and JunD) and Fos protein family (c-Fos, FosB, Fra-1, and Fra-2) [25]. However, c-Fos and c-Jun are the most common subunits of AP-1, which may be assembled as homo- or hetero-dimers of AP-1 [26,27]. Regulation of AP-1 subunits can be achieved via gene transcription, mRNA stability, and post-translational processing. The most important post-translational control is the phosphorylation induced by MAPK signaling pathways and cellular stress [28]. While c-Fos was rapidly and transiently upregulated and phosphorylated in 2-CE-treated astrocytes, both upregulation and phosphorylation of c-Jun occurred much later than c-Fos. In addition, as with NF-κB inhibition, blocking AP-1 also ameliorated MMP-9 overexpression in astrocytes treated by 2-CE, suggesting that activation of AP-1 was also involved in eliciting MMP-9 in 2-CE-treated astrocytes.

**Figure 9:**
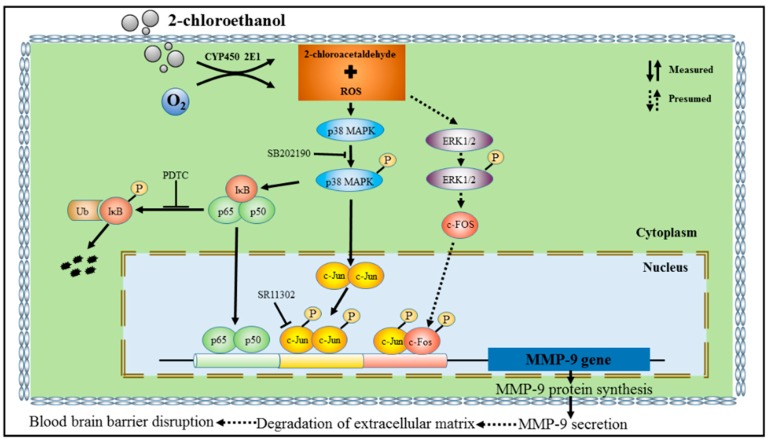
Schematic diagram.

The MAPK signal pathways are involved in a variety of fundamental cellular processes, among which the p38 MAPK pathway is involved in response to a wide range of extracellular stimuli, and plays a key role in the regulation of pro-inflammatory networks and biosynthesis of TNF-α, IL-1β, and MMP-9. Previous studies have shown that overexpression of MMP-9 elicited by inflammatory response in astrocytes could be regulated by the p38 MAPK signaling pathway [29]. Other studies indicated that MMP-9 expression could be modulated via activation of p38 MAPK signaling pathway in various cell types [30,31]. Therefore, we also explored the role of this pathway in the activation of NF-κB and AP-1 in 2-CE-treated astrocytes. Inhibition of p38 MAPK markedly reduced p65 overexpression and IκBα phosphorylation in 2-CE-treated astrocytes. Furthermore, the increased nuclear translocation of p65 and decreased IκBα protein levels in 2-CE-treated astrocytes were also ameliorated because of the suppressed phosphorylation of IκBα. Therefore, our results suggested that the p38 MAPK signaling pathway is involved in p65 expression and IκBα phosphorylation induced by 2-CE in astrocytes. In addition, p38 MAPK inhibition also markedly reduced the enhanced c-Jun expression and phosphorylation in 2-CE-treated astrocytes. As c-Fos upregulation and phosphorylation occurred ahead of p38 MAPK phosphorylation (levels of p-p38 in 2-CE-treated astrocytes increased as early as 2 h, and remained significantly high till 24 h, data as shown in Figure S1), we hypothesized that p38 MAPK signaling pathway was not involved in the regulation of c-Fos. Although the mechanisms by which c-Fos was upregulated and phosphorylated in 2-CE-treated astrocytes are still unclear, the ERK1/2 signaling pathway suggested by the recent studies might be involved in the immediately regulation of c-Fos [13]. The proposal schematic diagram was shown in Figure 9.

Taken together, our results suggested that MMP-9 expression was upregulated through p38 MAPK mediated activation of NF-κB and AP-1 in 2-CE-treated astrocytes. Our findings might provide novel clues for clarifying the mechanisms underlying 1,2-DCE associated cerebral edema.

## Figures and Tables

**Table 1:** Primer sequences for the target genes. MMP-9—matrix metalloproteinases-9; IκBα—inhibitor of κBα.

Gene	Primer Sequence (5′-3′)	
*MMP-9*	Forward	5′-ATCCGCAGTCCAAGAAGATT-3′
	Reverse	5′-GCCAGAGAACTCGTTATCCA-3′
*p38*	Forward	5′-CCGAGCGATACCAGAACCT-3′
	Reverse	5′-AACACATCCAACAGACCAATCA-3′
*p65*	Forward	5′-TTAGCCATCATCCACCTTC-3′
	Reverse	5′-AGTCCTCCACCACATCTT-3′
*IκBα*	Forward	5′-GAGGATTACGAGCAGATGG-3′
	Reverse	5′-ATGGTCAGTGTCTTCTCTTC-3′
*c-Jun*	Forward	5′-ACGACCTTCTACGACGAT-3′
	Reverse	5′-CATTGCTGGACTGGATGAT-3′
*c-Fos*	Forward	5′-TCCGAAGGGAAAGGAATAAG-3′
	Reverse	5′-AGTCAAGTCCAGGGAGGTC-3′
*GAPDH*	Forward	5′-GCAAGAGAGAGGCCCTCAG-3′
	Reverse	5′-TGTGAGGGAGATGCTCAGTG-3′

## Supplementary Materials

The following are available online at http://www.mdpi.com/2073-4409/7/8/96/s1, Figure S1: Alteration in expression and phosphorylation of p38 MAPK in 2-CE exposed rat astrocytes along with the exposure time and 2-CE concentrations.

## Abbreviations

1,2-DCE	1,2-dichloroethane
2-CE	2-chloroethanol
NF-κB	nuclear factor kappa B
IκBα	inhibitor of κBα
AP-1	activator protein-1
MMP-9	matrix metalloproteinase-9
MAPK	mitogen-activated protein kinase
PDTC	pyrrolidine dithiocarbamate
SR	SR11302
SB	SB202190

## References

[1] Liu J.R., Fang S., Ding M.P., Chen Z.C., Zhou J.J., Sun F. (2010). Toxic encephalopathy caused by occupational exposure to 1,2-Dichloroethane. J. Neurol. Sci..

[2] Zhang Q., Niu Q., Li L.Y., Yang L., Guo X.L., Huang J.X. (2011). Establishment of a poisoned animal model of toxic encephalopathy induced by 1,2-dichloroethane. Int. J. Immunopathol. Pharmacol..

[3] Chen S., Zhang Z., Lin H., Chen Z., Wang Z., Wang W. (2015). 1,2-Dichloroethane-induced toxic encephalopathy: a case series with morphological investigations. J. Neurol. Sci..

[4] Guengerich F.P., Crawford W.M., Domoradzki J.Y., Macdonald T.L., Watanabe P.G. (1980). In vitro activation of 1,2-dichloroethane bymicrosomal and cytosolic enzymes. Toxicol. Appl. Pharmacol..

[5] Igwe O.J., Que Hee S.S., Wagner W.D. (1986). Inhalation pharmacokinetics of 1,2-dichloroethane after different dietary pretreatments of male Sprague-Dawley rats. Arch. Toxicol..

[6] Raucy J.L., Kraner J.C., Lasker J.M. (1993). Bioactivation of halogenated hydrocarbons by cytochrome P4502E1. Crit. Rev. Toxicol..

[7] Wang T., Liao Y., Sun Q., Tang H., Wang G., Zhao F., Jin Y. (2017). Upregulation of Matrix Metalloproteinase-9 in Primary Cultured Rat Astrocytes Induced by 2-Chloroethanol Via MAPK Signal Pathways. Front. Cell. Neurosci..

[8] Wang G., Yuan Y., Zhang J., Gao L., Tan X., Yang G., Lv X., Jin Y. (2014). Roles of aquaporins and matrix metalloproteinases in mouse brain edema formation induced by subacute exposure to 1,2-dichloroethane. Neurotoxicol. Teratol..

[9] Haorah J., Ramirez S.H., Schall K., Smith D., Pandya R., Persidsky Y. (2007). Oxidative stress activates protein tyrosinkinase and matrix metalloproteinases leading to blood-brain barrier dysfunction. J. Neurochem..

[10] Rosenberg G.A. (2002). Matrix metalloproteinases in neuroinflammation. Glia.

[11] Rosell A., Ortega-Aznar A., Alvarez-Sabín J., Fernández-Cadenas I., Ribó M., Molina C.A., Lo E.H., Montaner J. (2006). Increased brain expression of matrix metalloproteinase-9 after ischemic and hemorrhagic human stroke. Stroke.

[12] Sun Q., Liao Y., Wang T., Tang H., Wang G., Zhao F., Jin Y. (2017). 2-Chloroethanol induced upregulation of matrix metalloproteinase-2 in primary cultured rat astrocytes via MAPK signal pathways. Front. Neurosci..

[13] Wang H.H., Hsieh H.L., Wu C.Y., Sun C.C., Yang C.M. (2009). Oxidized low-density lipoprotein induces matrix metalloproteinase-9 expression via a p42/p44 and JNK-dependent AP-1 pathway in brain astrocytes. Glia.

[14] Speidl W.S., Kastl S.P., Hutter R., Katsaros K.M., Kaun C., Bauriedel G., Maurer G., Huber K., Badimon J.J., Wojta J. (2011). The complement component C5a is present in human coronary lesions in vivo and induces the expression of MMP-1 and MMP-9 in human macrophages in vitro. FASEB J..

[15] Hwang Y.P., Yun H.J., Choi J.H., Han E.H., Kim H.G., Song G.Y., Kwon K.I., Jeong T.C., Jeong H.G. (2011). Suppression of EGF-induced tumor cell migration and matrix metalloproteinase-9 expression by capsaicin via the inhibition of EGFR-mediated FAK/Akt, PKC/Raf/ERK, p38 MAPK, and AP-1. Mol. Nutr. Food Res..

[16] Park J., Kwak C.H., Ha S.H., Kwon K.M., Abekura F., Cho S.H., Chang Y.C., Lee Y.C., Ha K.T., Chung T.W. (2018). Ganglioside GM3 suppresses lipopolysaccharide-induced inflammatory responses in rAW 264.7 macrophage cells through NF-κB, AP-1, and MAPKs signaling. J. Cell. Biochem..

[17] Hsieh C.C., Papaconstantinou J. (2004). Akt/PKB and p38 MAPK signaling, translational initiation and longevity in Snell dwarf mouse livers. Mech. Ageing Dev..

[18] Wu C.Y., Hsieh H.L., Jou M.J., Yang C.M. (2004). Involvement of p42/p44 MAPK, p38 MAPK, JNK and nuclear factor-kappa B in interleukin-1beta-induced matrix metalloproteinase-9 expression in rat brain astrocytes. J. Neurochem..

[19] Herbein G., Varin A., Fulop T. (2006). NF-kB, AP-1, Zinc-deficiency and aging. Biogerontology.

[20] Gosselin K., Abbadie C. (2003). Involvement of Rel/NF-kappa B transcription factors in senescence. Exp. Gerontol..

[21] Liu X., Kumar A. (2015). Differential signaling mechanism for HIV-1 Nef-mediated production of IL-6 and IL-8 in human astrocytes. Sci. Rep..

[22] Shih R.H., Wang C.Y., Yang C.M. (2015). NF-kappaB Signaling Pathways in Neurological Inflammation: A Mini Review. Front. Mol. Neurosci..

[23] Carter A.B., Knudtson K.L., Monick M.M., Hunninghake G.W. (1999). The p38 mitogen-activated protein kinase is required for NF-kappa B-dependent gene expression. The role of TATA-binding protein (TBP). J. Biol. Chem..

[24] Huang C.W., Feng W., Peh M.T., Peh K., Dymock B.W., Moore P.K. (2016). A novel slow-releasing hydrogen sulfide donor, FW1256, exerts anti-inflammatory effects in mouse macrophages and in vivo. Pharmacol. Res..

[25] Ding J., Huang Y., Ning B., Gong W., Li J., Wang H., Chen C.Y., Huang C. (2009). TNF-alpha induction by nickel compounds is specific through ERKs/AP-1-dependent pathway in human bronchial epithelial cells. Curr. Cancer Drug Targets.

[26] Karin M., Liu Z., Zandi E. (1997). AP-1 function and regulation. Curr. Opin. Cell Biol..

[27] Shaulian E., Karin M. (2002). AP-1 as a regulator of cell life and death. Nat. Cell Biol..

[28] Bogoyevitch M.A., Kobe B. (2006). Uses for JNK: The many and varied substrates of the c-Jun N-terminal kinases. Microbiol. Mol. Biol. Rev..

[29] Miraglia M.C., Scian R., Samartino C.G., Barrionuevo P., Rodriguez A.M., Ibañez A.E., Coria L.M., Velásquez L.N., Baldi P.C., Cassataro J. (2013). Brucella abortus induces TNF-α-dependent astroglial MMP-9 secretion through mitogen-activated protein kinases. J. Neuroinflamm..

[30] Lappas M., Riley C., Lim R., Barker G., Rice G.E., Menon R., Permezel M. (2011). MAPK and AP-1 proteins are increased in term pre-labour fetal membranes overlying the cervix: Regulation of enzymes involved in the degradation of fetal membranes. Placenta.

[31] Lian S., Xia Y., Khoi P.N., Ung T.T., Yoon H.J., Kim N.H., Kim K.K., Jung Y.D. (2015). Cadmium induces matrix metalloproteinase-9 expression via ROS-dependent EGFR, NF-κB, and AP-1 pathways in human endothelial cells. Toxicology.

