# Supplementary material for: Association of NF-κB and AP-1 with MMP-9 Overexpression in 2-Chloroethanol Exposed Rat Astrocytes
Source: Cells. 2018 Aug 7;7(8):96. doi: 10.3390/cells7080096 (PMC6115792; doi:10.3390/cells7080096)
Supplement: Supplementary file 1 [file cells-07-00096-s001.pdf]

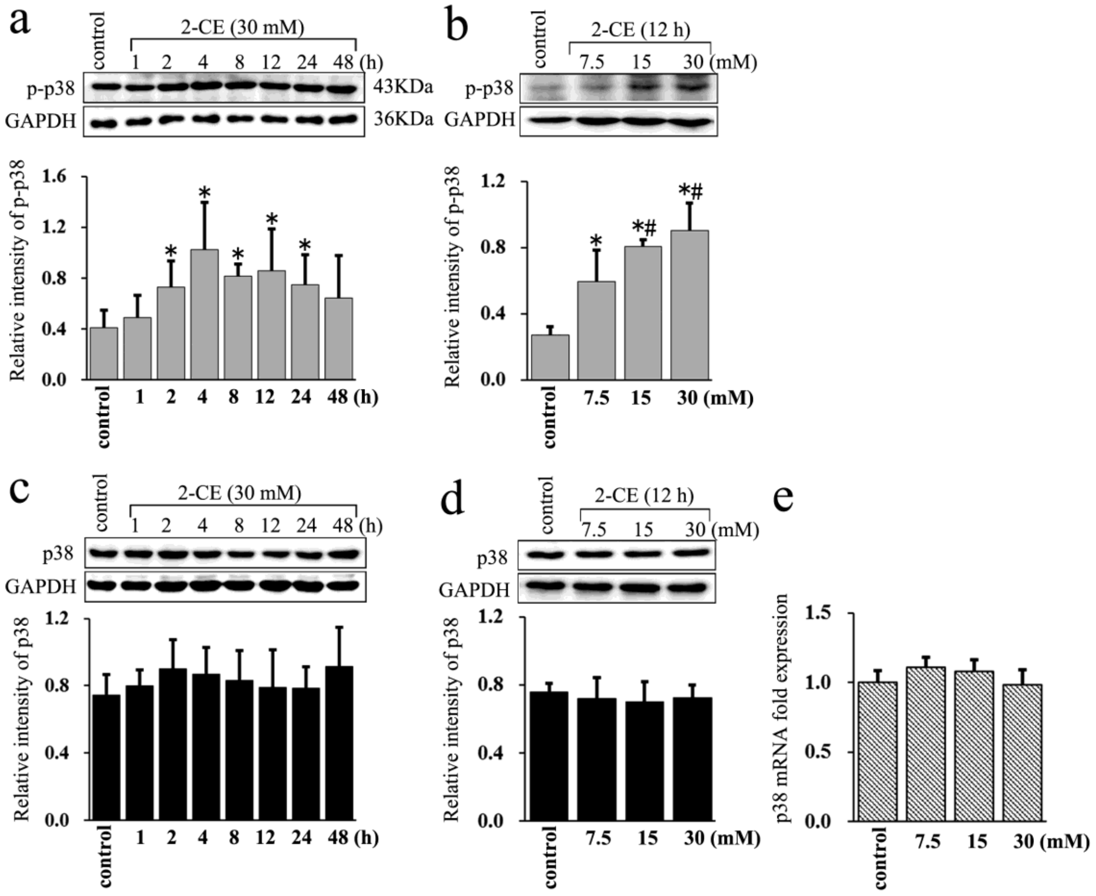

Figure S1 Alteration in expression and phosphorylation of p38 MAPK in 2-CE exposed rat astrocytes along with the exposure time and 2-CE concentrations
